# Assessing the quality of internet resources on pediatric neurosurgery for caregivers

**DOI:** 10.1007/s00381-026-07510-7

**Published:** 2026-11-05

**Authors:** Paulina Günzerodt, Janek Garber, Julia Bettina Kräutler, Daniel Monden, Mohammad Hazaymeh, Daniel Dubinski, Dorothee Mielke, Fabian B. Fahlbusch, Peter Baumgarten

**Affiliations:** 1 Department of Neurosurgery, Faculty of Medicine, University of Augsburg, Stenglinstraße 2, 86156 Augsburg, Germany; 2 Department of Neurosurgery, University Hospital Jena, Friedrich Schiller University, Jena, Germany; 3 Department of Neurosurgery, University Medicine Rostock, Rostock, Germany; 4 Section of Neonatology and Pediatric Intensive Care, Department of Pediatrics and Adolescent Medicine, Faculty of Medicine, University of Augsburg, Augsburg, Germany

**Keywords:** Online information, DISCERN, JAMA, Health literacy

## Abstract

**Purpose:**

Parents increasingly seek online information about pediatric neurosurgical conditions. We assessed the quality and transparency of German- and English-language pages on seven common conditions.

**Methods:**

A simulated lay Google search (30 December 2025) used one German and one English term per condition. The first 20 results per query were screened; the condition-website record was the unit of analysis. Two raters independently scored each page with the 16-item DISCERN Plus instrument, an adapted DISCERN version with condition-specific content items (range 16–80), and the four JAMA benchmarks. Analyses used non-parametric tests and mixed models accounting for domain clustering.

**Results:**

After five exclusions, 275 records remained (139 English, 136 German). The mean DISCERN Plus score was 39.9 ± 12.3 of 80; 46.9% fell into the exploratory “poor” or “very poor” bands. The mean JAMA score was 2.6 ± 1.0, and 54 pages (19.6%) met all four benchmarks. English pages scored higher (DISCERN Plus median 41.0 vs 35.8, *p* < 0.001; JAMA 3.0 vs 2.0, *p* = 0.002), also after adjustment for host type and domain clustering (+ 4.6 points, *p* = 0.010). Scores differed by host type in the overall test, with descriptively the highest medians for governmental sites; no differences between conditions were detected. Search rank was weakly associated with DISCERN Plus scores and not significantly with JAMA scores.

**Conclusions:**

Information for caregivers is of moderate-to-low quality with marked transparency deficits, and Google ranking is only weakly related to quality. Institutions should pilot a pre-publication checklist for family-facing pages, and clinicians should recommend vetted resources.

**Supplementary Information:**

The online version contains supplementary material available at https://doi.org/10.1007/s00381-026-07510-7.

## Introduction

Pediatric neurosurgical disorders comprise congenital and acquired conditions that often present in early childhood and can affect neurodevelopment, cognition, and quality of life [1, 2]. Although functional outcomes have improved considerably, parents confronted with a new diagnosis in their child frequently experience uncertainty and distress and seek information beyond the clinical consultation, most often on the internet, in order to understand the condition, the anticipated treatment course and the long-term prognosis [3–5].

The quality of online medical information is highly variable. Evaluations in pediatric and surgical fields using DISCERN and the JAMA benchmarks have repeatedly found patient-oriented resources to be incomplete, poorly referenced, commercially biased, or hard to understand [6–9]. In neurosurgery, YouTube content on hydrocephalus and craniosynostosis was rated poor despite hospital and educational authorship [10, 11], and although high-quality written material exists, families may not find it because of ranking algorithms and competing low-quality pages [12]. Pediatric neuro-oncology websites show comparable deficits and reading levels above the recommended lay level [13–15]. Parents rarely discuss their findings with the treating physician [16, 17].

In pediatric neurosurgery, where decisions involve high-risk procedures with potentially lifelong consequences, misleading information may delay presentation, foster unrealistic expectations, or undermine adherence. Systematic quality data for this field, and particularly across languages, remain scarce.

Extending a previous evaluation of German- and English-language information on neonatal jaundice [18], we assessed pages on seven common pediatric neurosurgical conditions with the DISCERN Plus instrument, an adapted version of DISCERN, and the JAMA benchmarks, and examined differences by language, condition, host type, and search rank.

## Methods

### Study design

This cross-sectional study evaluated publicly accessible web pages providing lay information on common pediatric neurosurgical conditions. Only previously published content was analyzed, so ethics approval was not required. The unit of analysis was the condition-website record, that is, one page evaluated in the context of one search term. A page retrieved for two conditions would have been rated separately for each, whereas repeated retrieval of the same page within one query or language was treated as a duplicate.

### Selection of pediatric neurosurgical conditions and search terms

Seven entities were selected for their frequency, their representation in patient education material and their relevance for parental decision-making, covering the domains that dominate pediatric neurosurgical outpatient work: cerebrospinal fluid disorders, craniofacial and dysraphic malformations, and the most frequent posterior fossa and sellar tumors of childhood. They span a wide incidence spectrum, from hydrocephalus, the most frequent pediatric neurosurgical condition worldwide [19], to craniopharyngioma [20]. Chiari malformation and tethered cord were included although both can also present in adulthood: Chiari malformation type I is found in up to 3.6% of children undergoing cranial or cervical MRI and its decompression is among the most common pediatric neurosurgical operations [21, 22], and tethered cord syndrome is predominantly diagnosed and operated on in childhood [23]. Apart from hydrocephalus, where the lay term specified children, pages were not required to address children explicitly. One English and one German lay term were predefined per condition: *Hydrocephalus child/Hydrozephalus Kind*, *craniosynostosis/Kraniosynostose*, *Chiari malformation/Chiari Malformation*, *Tethered cord/Tethered Cord*, *Medulloblastoma/Medulloblastom*, *Craniopharyngioma/Kraniopharyngeom*, *pilocytic astrocytoma/pilozytisches Astrozytom*. Other relevant conditions, including spina bifida, spinal dysraphism, neurovascular malformations, epilepsy surgery, and pediatric neurotrauma, belong to further topic areas of the same search protocol that are reported separately (see Declarations, related manuscripts). Terms were not derived from search-volume statistics or validated with caregivers, which is acknowledged as a limitation; the search simulates a lay search for these conditions rather than documented caregiver behavior.

### Search strategy

The search and extraction of result lists were performed with Google on 30 December 2025 between 9 and 11 am Central European Time. Each page was archived as a PDF on the day of its rating, between 22 February and 7 March 2026.

A virtual private network set the search location to Berlin, Germany, for German queries and to New York, USA, for English queries. Cache, cookies, and history were cleared before each session, private mode was used, and language and region filters were set accordingly.

For each of the 14 queries (7 conditions × 2 languages), the first 20 organic results were recorded in their original order, yielding 280 condition-website records; sponsored and clearly labeled paid results were noted separately and not rated. The search rank of each record is its position in this original list; records excluded afterwards (duplicates, paywalls) were removed without renumbering the remaining records.

### Inclusion and exclusion criteria

Websites were eligible for inclusion if they provided substantive information (≥ 1 paragraph of continuous text) on at least one of the target pediatric neurosurgical conditions and were freely accessible without registration or payment.

Exclusion criteria were duplicate or mirror pages, purely technical content for professionals, non-text formats without explanatory text, pages behind paywalls or with repeated server unavailability, and forums or social media posts without a stable information page. Duplicates were defined a priori as records pointing to identical content, including the same content under two URLs and pages appearing in one language result list after having been captured in the other. The complete list of inclusion and exclusion criteria was specified in the internal study protocol of the search series before the search was run. The rating chart (Supplementary File S1) lists only the four page-level criteria that were ticked at the time of rating (less than one paragraph of information on the search topic, password restriction, repeated server unavailability, and direct forwarding to another domain); the remaining criteria (duplicates, purely technical professional content, non-text formats, paywalls, forums, and social media posts) were applied when the result lists were screened. Both lists are reproduced in Supplementary File S1.

Five of the 280 records were excluded: three duplicates and two paywalled pages. The duplicates were two English-language pages (Mayo Clinic and NHS, Chiari malformation) that reappeared in the German result list and one German guideline (AWMF S2k, childhood hydrocephalus) listed both as a PDF and as a registry detail page, of which the PDF was retained. The paywalled pages were a medulloblastoma review and a tethered cord portal page. No record was excluded for insufficient text, leaving 275 records. The retained AWMF guideline is addressed primarily to physicians but met the inclusion criteria and none of the predefined exclusion criteria, which cover purely technical content but not professional documents that an informed layperson can read; it was therefore kept.

### Categorization by host type and language

Host type was assigned by consensus of both raters from the operator stated in the imprint or “about us” section rather than the top-level domain, using five predefined categories: university/medical center, governmental organization, interest group or non-governmental organization (NGO), non-profit educational portal, and medical news site. A residual category (“other”) on the rating chart was not required after final allocation.

Language was documented as German or English on the basis of the primary text content. Where parallel language versions existed, the version corresponding to the search language was evaluated.

### Quality assessment tools

#### DISCERN Plus instrument

Websites were evaluated with the DISCERN Plus instrument, an adapted version of the DISCERN questionnaire for written consumer health information on treatment choices [24] that was developed for a series of evaluations of German- and English-language web information [18, 25–27] and was applied here in the form published by Baumgartner et al. [18], with the exception that item 16 is included in the total. The rating chart is provided in Supplementary File S1. Section 1 (items 1–8, reliability of the publication) is identical to the original DISCERN. In section 2, the seven treatment-related items of the original (how each treatment works, benefits, risks, consequences of no treatment, effect on quality of life, more than one treatment choice, support for shared decision-making) are replaced by seven condition-specific content items: consequences or risks of the condition (item 9), when to contact a physician (10), treatment options and their mechanism (11), risks of treatment (12), diagnostic procedure (13), effect of treatment on quality of life or treatment setting (14), and pre- or post-treatment recommendations (15). Consequently, the adapted instrument does not assess whether the benefits of treatment are described, whether it is made clear that there may be more than one treatment choice, or whether the page supports shared decision-making; these elements of the original DISCERN are not captured by the DISCERN Plus total. The rationale is that lay information on a disease entity, unlike the treatment leaflets DISCERN was written for, covers the condition, its diagnosis and its course as well as the therapy, so that several original items are not applicable where surveillance rather than a treatment decision predominates. Item 16, the overall rating of the publication, is retained from the original instrument. Each item is scored from 1 (definitely no) to 5 (definitely yes); all 16 items were summed, giving a total of 16–80 points. The adapted instrument showed substantial interrater agreement previously [18] and in the present study, but it has not been formally validated, and the validation of the original DISCERN does not transfer to the adapted section 2. The DISCERN Plus total is therefore treated as a continuous measure of the quality of the information process and presentation; like DISCERN itself, it does not assess clinical accuracy, a limitation that the DISCERN handbook states explicitly [24, 28]. For descriptive purposes, totals were additionally classified with the five-band scheme of Nghiem et al. [29] for the 16–80 range as applied by Valencia et al. [30] (16–26 “very poor,” 27–38 “poor,” 39–50 “fair,” 51–62 “good,” and 63–80 “excellent”). These thresholds were derived for the original instrument; their application to the adapted score is exploratory and the band frequencies are a descriptive summary of the score distribution, not validated quality categories. Because the primary score was the mean of two raters and can take half-point values, band limits were applied as upper bounds.

#### JAMA benchmarks

The four JAMA benchmarks—authorship, attribution, disclosure, and currency [31]—were each scored as present (1) or absent (0), giving a maximum of 4 points per website. Only rater-level totals were recorded, so the frequency of individual missing benchmarks cannot be reported. In the “Results” section, a page is described as having met all four benchmarks when both raters awarded all four (mean score 4.0), and as having met two or fewer benchmarks when the mean of the two ratings was 2.0 or lower. Because DISCERN Plus items 4 (sources of information) and 5 (currency) address the same page features as the JAMA attribution and currency benchmarks, the two instruments are not independent and a positive correlation between them is in part structural.

### Raters and interrater reliability

Two raters with clinical experience in pediatric neurosurgery rated all websites independently after joint calibration on a training set not included in the sample. All analyses, quality categories and figures are based on the arithmetic mean of the two ratings. No consensus rating was generated and no rater served as reference. Both raters are native German speakers with professional working proficiency in English.

Reliability was quantified with the two-way random-effects, single-rater, absolute-agreement intraclass correlation coefficient (ICC [A,1]), Spearman correlations, and absolute rater differences, interpreted according to Koo and Li [32].

### Statistical analysis

Descriptive statistics were calculated overall and by language, condition, and host category. The continuous DISCERN Plus total and the JAMA score were the primary outcomes; the classification of DISCERN Plus totals into the five bands was descriptive and exploratory. Because Shapiro–Wilk testing indicated non-normal distributions, groups were compared with Mann–Whitney *U* tests (language) and Kruskal–Wallis *H* tests (conditions, host types), with rank-biserial correlation (*r*_*rb*) and eta squared based on the *H* statistic (*η*^2^_*H* = (*H* − *k* + 1)/(*n* − *k*), with k groups) as effect sizes. Spearman correlations were used for search rank, overall and within each language, and between instruments. Because several domains contributed more than one record, three sensitivity analyses addressed non-independence: restriction to the first record per domain, domain-level means, and linear mixed models with a random intercept for domain and language and host type as fixed effects. The mixed models were sensitivity analyses to the non-parametric primary comparisons; for the JAMA score, a bounded count of 0–4 benchmarks, the linear mixed model is an approximation and is interpreted alongside the concordant non-parametric results. The language comparison was repeated within each host category and after exclusion of governmental sites; these comparisons, like those between conditions, were exploratory, and for the eight within-host tests (four host categories × two instruments), a Bonferroni-corrected threshold of *p* < 0.006 is given for orientation. Rank correlations were not adjusted for domain clustering. For the host-type comparisons, only the overall Kruskal–Wallis test was performed; no pairwise post hoc tests between host categories were conducted, and differences between individual host types are reported descriptively on the basis of medians and interquartile ranges. Two-sided *p* < 0.05 was considered significant. Analyses used JMP Edition 19 (JMP Statistical Discovery LLC, Cary, NC, USA). Generative artificial intelligence (Perplexity AI) was used for language editing and for assistance with the plotting scripts of the figures, as detailed in the “Declarations” section.

## Results

### Website sample and host characteristics

Of the 280 retrieved records, five were excluded (see Methods), leaving 275 condition-website records (139 English, 136 German). Each condition contributed 20 English and 20 German records except Chiari malformation (20 and 18), hydrocephalus and tethered cord (20 and 19 each), and medulloblastoma (19 and 20). Host categories were university or medical center (*n* = 137, 49.8%), educational (*n* = 59, 21.5%), interest group/NGO (*n* = 38, 13.8%), medical news (*n* = 22, 8.0%), and governmental (*n* = 19, 6.9%). Governmental sources were almost exclusively English (17 of 19), whereas educational and medical-news pages were more often German (Table 1).

**Table 1:** Sample characteristics by host type and language

*Host type*	*English, n (%)*	*German, n (%)*	*All, n (%)*
*University/medical center*	75 (54.0)	62 (45.6)	137 (49.8)
*Educational*	20 (14.4)	39 (28.7)	59 (21.5)
*Interest group/NGO*	21 (15.1)	17 (12.5)	38 (13.8)
*Medical news*	6 (4.3)	16 (11.8)	22 (8.0)
*Governmental*	17 (12.2)	2 (1.5)	19 (6.9)
*All*	139 (100.0)	136 (100.0)	275 (100.0)

### Overall quality and transparency

Across the 275 records the mean DISCERN Plus score was 39.9 ± 12.3 of 80 points (median 39.0, IQR 31.0–48.3; range 16–75). In the exploratory band classification, eight records (2.9%) were “excellent,” 49 (17.8%) “good,” 89 (32.4%) “fair,” 92 (33.5%) “poor,” and 37 (13.5%) “very poor,” so 129 (46.9%) fell into the two lowest exploratory bands. The mean JAMA score was 2.6 ± 1.0 (median 2.5). Only 54 records (19.6%) met all four benchmarks according to both raters (mean score 4.0), whereas 118 (42.9%) had a mean score of 2.0 or lower, i.e., met two or fewer benchmarks. The instruments were moderately correlated (*ρ* = 0.531, *p* < 0.001).

### Language, condition, and host comparisons

**Fig. 1:**
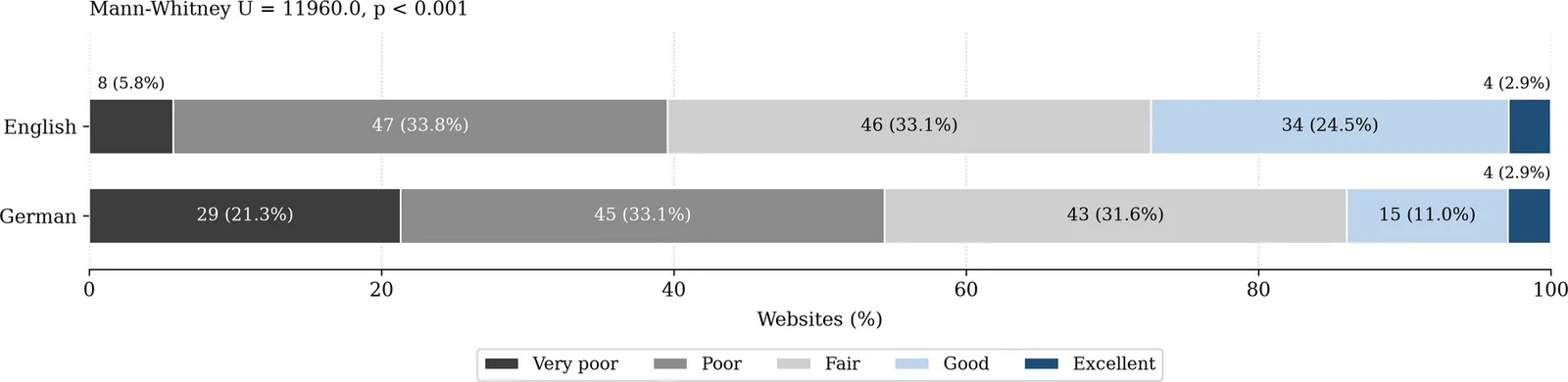
Distribution of DISCERN Plus quality categories by language. Exploratory quality bands follow Nghiem et al. for the 16–80 range of the 16-item DISCERN Plus score (16–26 “very poor,” 27–38 “poor,” 39–50 “fair,” 51–62 “good,” 63–80 “excellent”). “Very poor” ratings were markedly more common among German-language websites, whereas “good” ratings were more common among English-language websites. The Mann–Whitney test (*U* = 11,960.0, *p* < 0.001) compares the underlying continuous DISCERN Plus scores between languages, not the category proportions; the band classification is exploratory

English-language websites scored higher on both instruments. For DISCERN Plus, the median (IQR) was 41.0 (33.5–51.0) versus 35.8 (28.4–45.5) (*U* = 11,960.0, *p* < 0.001; *r*_*rb* = 0.27) for JAMA 3.0 (2.0–3.5) versus 2.0 (1.5–3.0) (*U* = 11,504.5, *p* = 0.002; *r*_*rb* = 0.22). After adjustment for host type and domain clustering, English pages scored 4.6 DISCERN Plus points higher (95% CI 1.1–8.1, *p* = 0.010; see below). “Very poor” ratings were more common among German pages (29, 21.3%) than English pages (8, 5.8%), “good” ratings more common in English (34, 24.5%) than German records (15, 11.0%; Fig. 1).

**Fig. 2:**
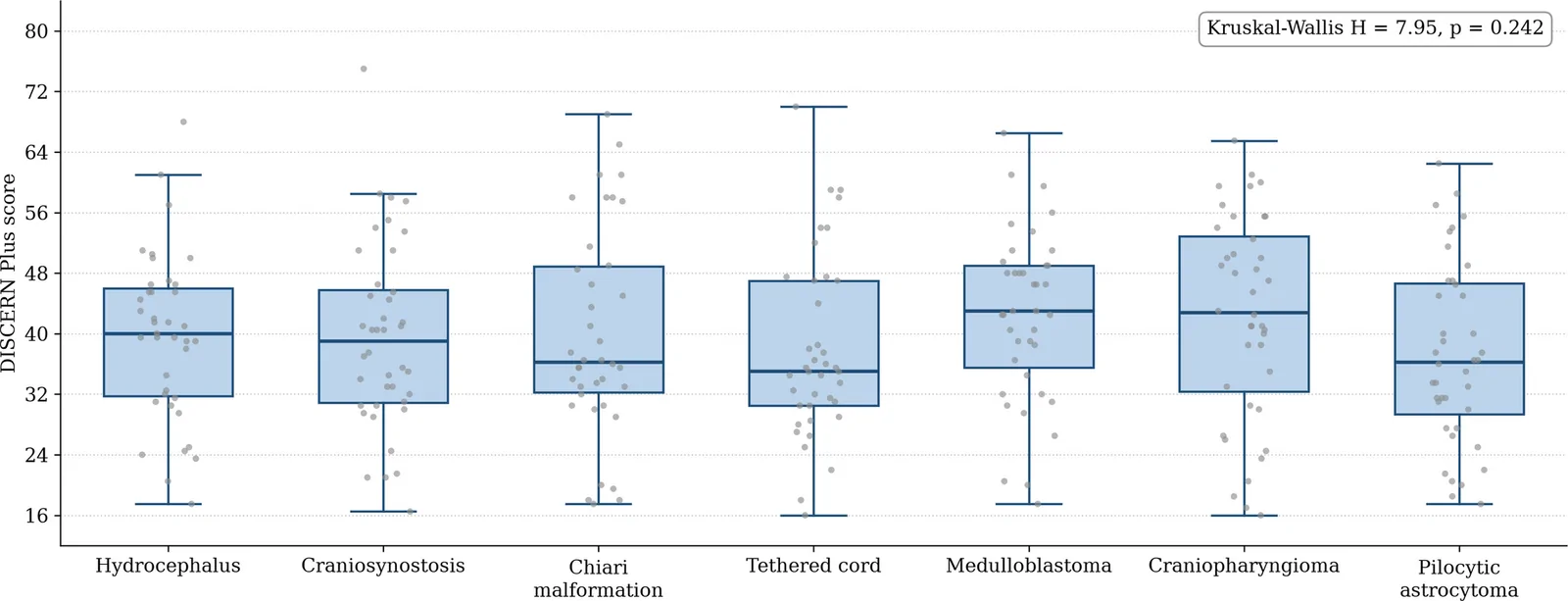
Distribution of mean DISCERN Plus total scores by pediatric neurosurgical condition. Boxes show the median and interquartile range; whiskers show the 1.5 × IQR range; dots indicate individual condition-website records. Differences across conditions were not statistically significant (Kruskal–Wallis *H* = 7.95, *p* = 0.242)

**Fig. 3:**
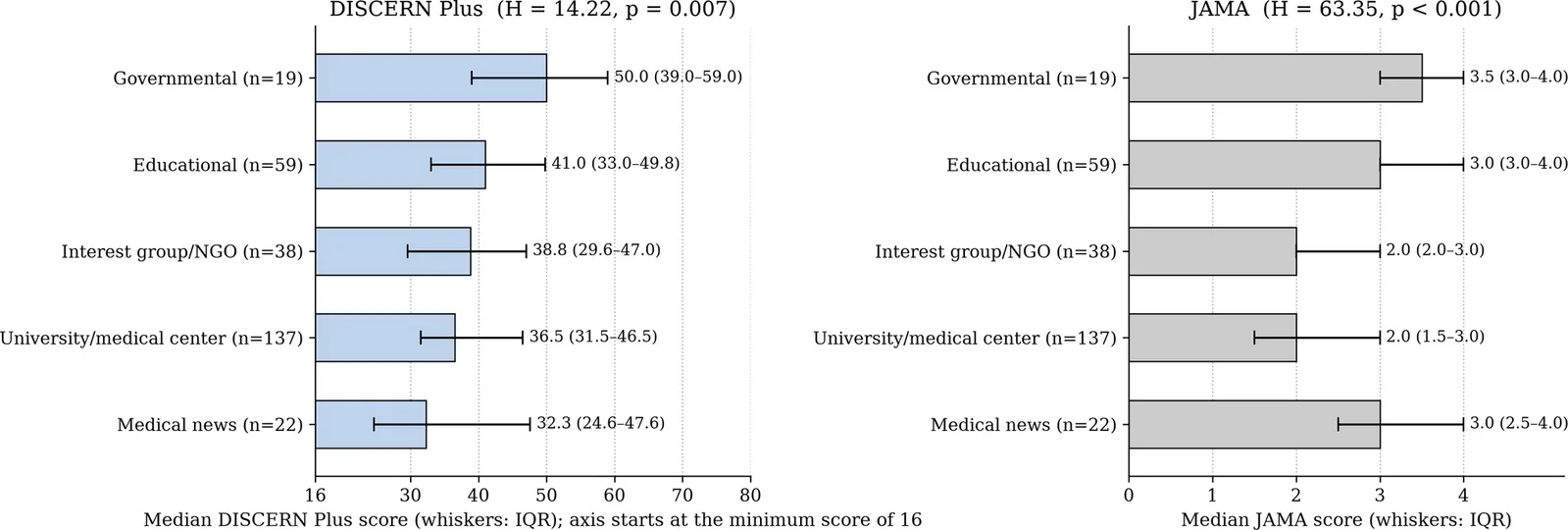
DISCERN Plus and JAMA scores by host type. Governmental hosts displayed the highest median scores; the host-type effect was strongest for JAMA transparency (Kruskal–Wallis *H* = 63.35, *p* < 0.001). Bars show the median score per host type, whiskers the interquartile range, and labels give the median (IQR); the number of records per host type is given in the axis labels. The DISCERN Plus axis starts at 16, the minimum possible total score. The *p*-values refer to the overall Kruskal–Wallis tests; no pairwise post hoc tests between host types were performed, so differences between individual host types are descriptive

No statistically significant differences between the seven conditions were detected (DISCERN Plus *H* = 7.95, *p* = 0.242, *η*^2^_*H* = 0.007; JAMA *H* = 11.20, *p* = 0.083, *η*^2^_*H* = 0.019), with median DISCERN Plus values from 35.0 (tethered cord) to 43.0 (medulloblastoma; Fig. 2). Host type was associated with both outcomes in the overall tests: DISCERN Plus differed across hosts (*H* = 14.22, *p* = 0.007, *η*^2^_*H* = 0.038), and the effect was more pronounced for JAMA transparency (*H* = 63.35, *p* < 0.001, *η*^2^_*H* = 0.22). Descriptively, the DISCERN Plus median was highest for governmental (50.0) and lowest for medical-news sites (32.3), and the JAMA median was higher for governmental (3.5), educational (3.0), and medical-news pages (3.0) than for university/medical-center and interest group/NGO sources (2.0 each; Fig. 3, Tables 2 and 3). Pairwise post hoc tests between host categories were not performed, so these contrasts between individual host types are descriptive.

**Table 2:** DISCERN Plus and JAMA scores by language, condition, and host type

*Stratum*	*n*	*DISCERN Plus, median (IQR)*	*DISCERN Plus, mean* ± *SD*	*JAMA, median (IQR)*
*All websites*	275	39.0 (31.0–48.3)	39.9 ± 12.3	2.5 (2.0–3.5)
*Language*				
* English*	139	41.0 (33.5–51.0)	42.7 ± 11.4	3.0 (2.0–3.5)
* German*	136	35.8 (28.4–45.5)	37.0 ± 12.6	2.0 (1.5–3.0)
*Condition*				
* Hydrocephalus*	39	40.0 (31.8–46.0)	39.7 ± 11.0	2.0 (1.5–3.5)
* Craniosynostosis*	40	39.0 (30.9–45.8)	39.5 ± 12.3	2.5 (1.5–3.0)
* Chiari malformation*	38	36.3 (32.2–48.9)	39.9 ± 13.8	2.5 (1.5–3.0)
* Tethered cord*	39	35.0 (30.5–47.0)	37.9 ± 12.1	2.0 (1.5–3.0)
* Medulloblastoma*	39	43.0 (35.5–49.0)	42.4 ± 11.2	3.0 (2.0–3.8)
* Craniopharyngioma*	40	42.8 (32.4–52.9)	42.3 ± 13.5	3.0 (2.0–3.5)
* Pilocytic astrocytoma*	40	36.3 (29.4–46.6)	37.3 ± 12.0	3.0 (2.0–3.5)
*Host type*				
* University/medical center*	137	36.5 (31.5–46.5)	38.9 ± 11.1	2.0 (1.5–3.0)
* Educational*	59	41.0 (33.0–49.8)	40.6 ± 12.6	3.0 (3.0–4.0)
* Interest group/NGO*	38	38.8 (29.6–47.0)	38.9 ± 12.8	2.0 (2.0–3.0)
* Medical news*	22	32.3 (24.6–47.6)	36.2 ± 13.6	3.0 (2.5–4.0)
* Governmental*	19	50.0 (39.0–59.0)	50.2 ± 13.1	3.5 (3.0–4.0)

The 16-item DISCERN Plus total score ranges from 16 to 80 points; the score reported here is the arithmetic mean of two independent raters and can therefore take half-point values. JAMA score ranges from 0 to 4. *IQR*, interquartile range; *SD*, standard deviation

**Table 3:** Statistical comparisons

*Comparison*	*Metric*	*Test (statistic)*	*p-*value
*German vs English*	DISCERN Plus	Mann–Whitney *U* = 11,960.0 (*r*_*rb* = 0.27)	< 0.001
*German vs English*	JAMA	Mann–Whitney *U* = 11,504.5 (*r*_*rb* = 0.22)	0.002
*Across 7 conditions*	DISCERN Plus	Kruskal–Wallis *H* = 7.95 (*η*^2^_*H* = 0.007)	0.242
*Across 7 conditions*	JAMA	Kruskal–Wallis *H* = 11.20 (*η*^2^_*H* = 0.019)	0.083
*Across host types*	DISCERN Plus	Kruskal–Wallis *H* = 14.22 (*η*^2^_*H* = 0.038)	0.007
*Across host types*	JAMA	Kruskal–Wallis *H* = 63.35 (*η*^2^_*H* = 0.22)	< 0.001
*Google rank, all records*	DISCERN Plus	Spearman *ρ* = − 0.243	< 0.001
*Google rank, all records*	JAMA	Spearman *ρ* = − 0.116	0.055
*DISCERN Plus vs JAMA*	Score correlation	Spearman *ρ* = 0.531	< 0.001
*Google rank, English records*	DISCERN Plus	Spearman *ρ* = − 0.192	0.023
*Google rank, German records*	DISCERN Plus	Spearman *ρ* = − 0.316	< 0.001
*Google rank, English records*	JAMA	Spearman *ρ* = − 0.159	0.062
*Google rank, German records*	JAMA	Spearman *ρ* = − 0.096	0.265

### Non-independence of records and separation of language and host effects

The 275 records corresponded to 275 unique URLs on 148 domains; 58 domains contributed more than one record (maximum 8) and 185 records (67.3%) came from these domains. Restriction to the first record per domain (*n* = 148) preserved the language difference (DISCERN Plus *U* = 3412.5, *p* = 0.009; JAMA *U* = 3465.0, *p* = 0.004) and the host-type effect (DISCERN Plus *H* = 12.44, *p* = 0.014; JAMA *H* = 42.76, *p* < 0.001), and domain-level means gave equivalent results (*p* = 0.003 and *p* < 0.001). In mixed models with a random intercept for domain, English pages scored 5.6 DISCERN Plus points higher (95% CI 2.2–9.0, *p* = 0.001) and 4.6 points higher after adjustment for host type (95% CI 1.1–8.1, *p* = 0.010); governmental hosts scored 10.6 points higher than university hosts (95% CI 3.8–17.4, *p* = 0.002). For JAMA, English language remained associated with a higher score (+ 0.50 benchmarks, 95% CI 0.24–0.75, *p* < 0.001), as did educational, governmental, and medical-news hosts (all *p* < 0.001).

In exploratory within-host comparisons, the DISCERN Plus language effect was significant among educational portals (median 49.3 vs 40.0, *p* = 0.001) and pointed in the same direction among university/medical-center (39.0 vs 35.3, *p* = 0.073) and medical-news pages (*p* = 0.121), with no difference for interest group/NGO pages (*p* = 0.78). For JAMA, it was most evident among university/medical-center (*p* < 0.001) and medical-news pages (*p* = 0.041). Only the educational-portal DISCERN Plus difference and the university/medical-center JAMA difference remain below the Bonferroni-corrected threshold of *p* < 0.006. Excluding governmental sites, the difference persisted for both instruments (DISCERN Plus *p* = 0.001; JAMA *p* = 0.014). Details are given in Supplementary Table S1 and Supplementary Figure S1.

### Search rank association

Google search rank correlated weakly and inversely with DISCERN Plus (*ρ* = − 0.243, *p* < 0.001), that is, higher-ranked results scored slightly better, in both languages (English *ρ* = − 0.192, *p* = 0.023; German *ρ* = − 0.316, *p* < 0.001). For JAMA, the correlations were not significant (*ρ* = − 0.116, *p* = 0.055). These correlations describe a weak monotonic association and were not adjusted for domain clustering.

### Interrater reliability

Agreement was good: for DISCERN Plus totals, the ICC (A,1) was 0.876 (95% CI 0.85–0.90) with a mean absolute difference of 5.3 points and for JAMA totals 0.829 (95% CI 0.79–0.86) with a mean absolute difference of 0.35 points (Table 4). Agreement indicates the reproducibility of the ratings, not the validity of the adapted instrument.

**Table 4:** Interrater reliability

*Score*	*ICC (A,1) (95% CI)*	*Mean absolute difference*	*Median absolute difference*	*Spearman ρ*
*DISCERN Plus total*	0.876 (0.85—0.90)	5.33	5	0.87
*JAMA total*	0.829 (0.79—0.86)	0.35	0	0.84

## Discussion

This standardized cross-language assessment of 275 condition-website records covering seven common pediatric neurosurgical conditions found online information for caregivers to be of moderate-to-low quality and transparency. With a mean DISCERN Plus score of 39.9 of 80, 46.9% of pages rated “poor” or “very poor” in the exploratory band classification and only about one in five meeting all four JAMA benchmarks, the findings are in line with earlier reports from pediatric neuro-oncology and general pediatric neurosurgery [7, 8, 13, 14]. A direct numerical comparison with studies that used the original DISCERN is limited, however, because section 2 of the DISCERN Plus chart differs from the treatment items of the original instrument; comparability is closest with the previous DISCERN Plus evaluations, in which German-language pages also scored lower than English-language pages [18, 27].

The language difference persisted after accounting for domain clustering and host-type composition (adjusted difference 4.6 DISCERN Plus points, 95% CI 1.1–8.1), which we regard as the most informative estimate of the language effect. Part of it is structural: governmental portals, which scored highest, were almost exclusively English (17 of 19), reflecting centrally curated services such as the NHS and MedlinePlus, for which no equivalent single-entry pediatric resource exists in German. Host composition alone does not explain the gap since it persisted after exclusion of governmental sites and in adjusted models, and was most evident among educational portals. German-language information is instead distributed across hospital departments, physician-facing reference works, and commercial health portals [6, 9, 18]. Because caregivers search predominantly in their primary language, this imbalance is clinically relevant. Search localization and health-system structures may contribute as well, so the comparison should be read as one between two information ecosystems rather than between languages per se.

The moderate correlation between the instruments indicates that sites which were more transparent also tended to receive higher DISCERN Plus scores. Part of this correlation is structural, because DISCERN Plus items 4 and 5 overlap with the JAMA attribution and currency benchmarks, so the association should not be read as evidence that transparent pages provide more balanced content. Host type was most strongly associated with transparency (*η*^2^_*H* 0.22 for JAMA versus 0.04 for DISCERN Plus), suggesting that hosts differ more in disclosure practice than in the substance of what they write. Descriptively, governmental and educational sites had the highest medians, whereas university and medical-center pages were heterogeneous despite their clinical authority and had a low median transparency score (2.0 of 4); interest groups and NGOs also had a low JAMA median, consistent with advocacy content being informative yet under-referenced. As no pairwise post hoc tests were performed, these contrasts between individual host types are descriptive observations rather than tested differences. Since item-level JAMA data were not retained, we cannot say which benchmark was missing most often. Both instruments assess transparency and the quality of the information process, not factual accuracy or guideline concordance [24]; a well-referenced, balanced page can still be outdated, so our results are not a judgement of clinical correctness. Academic departments should name authors and qualifications, cite sources, disclose sponsorship, and display dated review cycles.

The association between search rank and quality was statistically significant but weak (*ρ* = − 0.243) and not statistically significant for transparency (*ρ* = − 0.116, *p* = 0.055). Rank is therefore at best a weak guide to quality: caregivers relying on the first result page meet high- and low-quality content side by side, and excellent pages may rank far down [12, 33]. Ranking algorithms optimize for relevance and engagement rather than editorial quality.

Caregivers will continue to search online, most before or shortly after the first consultation [3–5]. The clinical question is what they find and whether it is discussed. Pediatricians who acknowledge the search and point families to reliable pages are perceived as supportive rather than dismissive [17], and institutional pages shape how parents judge what they read [34].

## Proposed measures for institutions and clinicians

Three measures are suggested by our data; they are proposals for evaluation, not validated standards. First, a short pre-publication quality check should become routine for institutional patient information. Our impression during rating, which was not quantified at item level, was that many deficits concerned recurring and easily correctable formal elements, so that a full DISCERN Plus rating would rarely be needed in practice. We therefore propose a 10-item checklist derived from the JAMA benchmarks, the reliability and content items of the DISCERN Plus chart, and the EQIP instrument [35, 36] (Supplementary Table S2). As a preliminary threshold, we suggest that a page should fulfill all four transparency items and at least four of the six content items before publication. This threshold was not validated in the present study and requires prospective evaluation, for example, by rating institutional pages before and after its introduction. Systematic use of such an instrument improved written material in a children’s hospital [35], whereas unstructured practice information averaged 39 of 100 points [36].

Second, the checklist should be embedded in an institutional workflow: a named clinical author and medical editor per condition page, a documented review interval of no more than 24 months, a standard footer carrying authorship, sources, sponsorship and review date, and inclusion of these pages in departmental quality management. The 24-month interval is a pragmatic choice rather than an evidence-based threshold: patient-information policies of health-care providers in the UK require clinical patient information to be reviewed every 2 years [37] or at least every 3 years [38], and we chose the shorter interval because web pages can be updated more easily than printed leaflets. Where resources are limited, adapting an existing high-quality resource under a clear license is more efficient than writing new content.

Third, the consultation should include a short information prescription: a one-page, condition-specific list of two to three vetted German- and English-language resources, stored in the electronic health record as a printable handout, together with an invitation to disclose previous searching (“What have you already read, and what worried you most?”). This turns an unstructured search into a supervised one at little cost in consultation time [17].

For the German-speaking area, the structural gap argues for a coordinated solution: a jointly maintained, condition-based caregiver portal under the auspices of the pediatric neurosurgery section of the German Society of Neurosurgery would address the missing central entry point more effectively than parallel departmental pages. Such a portal should in any case declare authorship, sources, sponsorship and review date, and the transparency elements of the JAMA benchmarks that were frequently missing in our sample. It should also name the available management options including watchful waiting and describe expected benefits, outcomes, and residual risks; these latter requirements were not assessed by our instrument and are derived from the treatment-choice items of the original DISCERN and from the EQIP criteria rather than from our results.

## Strengths and limitations

Strengths are the standardized cross-condition and cross-language design, two established instruments (the JAMA benchmarks and an adapted DISCERN version with documented interrater reliability), independent dual rating with good reliability (ICC [A,1] 0.83–0.88), pre-specified host categories, a reported exclusion process, and sensitivity analyses addressing domain clustering and the confounding of language by host type. With 275 records, the sample is among the larger evaluations in this field.

Several limitations apply. The search reflects a single time point (30 December 2025) and two VPN endpoints (Berlin and New York); because search algorithms are dynamic and partly personalized, other users may see different lists, and the rank correlations are a snapshot. Pages were rated between 22 February and 7 March 2026, so content may have changed after retrieval. Only the first 20 results and one lay term per condition and language were assessed, so pages ranked lower or found by other formulations may have been missed. The search terms were chosen as common lay designations and were not derived from search-volume data, so they may not fully reflect actual caregiver search behavior. The seven conditions were selected from a larger search protocol; other frequent entities such as spina bifida are reported separately, so the results do not cover the full spectrum of pediatric neurosurgery. Despite three sensitivity analyses, records from the same domain are not fully independent. The language comparison remains observational, as language, health system, search localization, and host composition are intertwined. We assessed static pages only, excluding multimedia platforms, social media, and conversational artificial intelligence. Readability was not formally assessed, although reading levels above the recommended lay level have been documented for comparable material [13, 15]. The main methodological limitation concerns the instrument: section 2 of the DISCERN Plus chart differs from the treatment items of the original DISCERN, so the adapted instrument is not validated for this application, its total is not equivalent to an original DISCERN total, and the quality bands were applied in an exploratory manner; good interrater agreement demonstrates reproducibility, not validity. Item-level DISCERN Plus and JAMA responses were not retained in the analysis dataset, so we cannot determine which items account for the observed differences, and neither instrument assesses clinical accuracy or guideline concordance [28]. In addition, because the treatment-choice items of the original DISCERN were replaced, the DISCERN Plus total does not reflect whether benefits of treatment, the existence of more than one treatment option, or support for shared decision-making are presented, so that our recommendations on these aspects rest on the original instruments rather than on our data [24]. The linear mixed model for the bounded JAMA score is an approximation; the rank correlations and within-host comparisons were not adjusted for domain clustering; and the absence of significant differences between conditions means that none was detected, not that the conditions are equivalent. Finally, host categorization required judgement for hybrid sites.

## Conclusions

Across seven common pediatric neurosurgical conditions, web-based information for caregivers is of moderate-to-low quality and transparency: nearly half of the pages (46.9%) fell into the two lowest bands of the exploratory classification and only about one in five met all four JAMA benchmarks. English-language pages scored higher, governmental resources had the highest median scores, and German-language and university or medical-center pages were heterogeneous and frequently missed transparency benchmarks; the language difference persisted after adjustment for host type and domain clustering. Because Google ranking is only weakly related to quality and not significantly related to transparency, institutions should pilot the proposed pre-publication checklist, pending its validation, for their family-facing pages, and clinicians should ask what families have read and hand out vetted resources.

## Supplementary Information

Below is the link to the electronic supplementary material.

ESM 1 (DOCX 20.2 KB)

ESM 2 (DOCX 280 KB)

## Data Availability

A pseudonymized record-level dataset (condition, search language, search rank, host category, and the DISCERN Plus and JAMA scores of both raters for each of the 275 records, with web domains replaced by a domain identifier) is available from the corresponding author on reasonable request.

## Declarations

### Declarations on the use of generative artificial intelligence

Perplexity AI (Perplexity AI Inc., San Francisco, CA, USA) was used for language editing of the human-written text and for assistance with the plotting scripts that generated the figures from the authors’ data. No scientific content, data or images were generated by artificial intelligence. The authors reviewed and edited all AI-assisted text and code, verified that it accurately reflects their original research, and take full responsibility for the content of the manuscript. All figures and tables are based on the authors’ original data.

### Related manuscripts

This study is part of a series of evaluations that use the same search protocol and rating chart for different fields of neurosurgery.
