# Supplementary material for: Assessing the quality of internet resources on pediatric neurosurgery for caregivers
Source: Childs Nerv Syst. 2026 Nov 5;42(1):412. doi: 10.1007/s00381-026-07510-7 (PMC13740078; doi:10.1007/s00381-026-07510-7)
Supplement: Supplementary file 1 [file 381_2026_7510_MOESM1_ESM.docx]

**Standardized Chart for the Quality Assessment of Websites**

Domain: ___________________________________

Google hit number: _______________

**Category**

| □ | Medical news site (sponsored) |
| --- | --- |
| □ | Medical center/university |
| □ | Interest group/NGO |
| □ | Governmental |
| □ | Educational |
| □ | Other |

**JAMA Benchmarks (yes/no) (Silberg et al. 1997)**

| □ | Authorship: authors/contributors/affiliations |
| --- | --- |
| □ | Attribution: References/sources/copyright |
| □ | Disclosure: ownership/sponsors/support |
| □ | Currency: creation/update date |

**Total JAMA score:** ______ / 4

**DISCERN Plus score (Baumgartner et al. 2024)**

**Section 1 – General quality of health information (1–5 points each)**

| **#** | **Item** | **Guidance** | **Pts** |
| --- | --- | --- | --- |
| 1 | **Are the aims clear?** | Are the objectives of the website clearly defined? What is the subject matter? Which topics are addressed? Who is the target audience? |  |
| 2 | **Does it achieve its aims?** | Does the website achieve its self-imposed goals? |  |
| 3 | **Is it relevant (reader's perspective)?** | Are questions addressed that readers would ask, using understandable language? |  |
| 4 | **Sources of information** | Are the information sources used clearly indicated? How many references are listed? Is the author clearly identified? |  |
| 5 | **Up-to-dateness of data** | Is the creation date, update date, and dates of references clearly indicated? |  |
| 6 | **Balanced and unbiased?** | Is the presentation balanced and objective? Are multiple sources cited? Is external review mentioned? |  |
| 7 | **Additional sources?** | Does the website contain information about supplementary aids and resources? |  |
| 8 | **Refer to areas of uncertainty?** | Are different possibilities addressed? Is lack of evidence mentioned? Is it indicated that information should not be used for self-diagnosis? |  |

Section 1 subtotal: ______ / 40

**Section 2 – Condition-specific questions (1–5 points each)**

| **#** | **Item** | **Guidance** | **Pts** |
| --- | --- | --- | --- |
| 9 | **Consequences/risks of [SEARCH TERM]** | Does the website describe the consequences or risks associated with [SEARCH TERM]? |  |
| 10 | **When to contact a physician** | Does the website provide recommendations on when to contact a physician regarding [SEARCH TERM]? |  |
| 11 | **Treatment options** | Does the website describe possible treatment options for [SEARCH TERM] and the mechanism of action of each? |  |
| 12 | **Risks of treatment** | Does the website describe the risks associated with each treatment procedure for [SEARCH TERM]? |  |
| 13 | **Diagnostic procedure** | Does the website describe the diagnostic procedure for [SEARCH TERM]? |  |
| 14 | **Quality of life / treatment setting** | Does the website describe how treatment for [SEARCH TERM] affects quality of life or the setting in which therapy is provided? |  |
| 15 | **Pre- or post-treatment recommendations** | Are recommendations made regarding pre- or post-treatment procedures for [SEARCH TERM]? |  |

Section 2 subtotal: ______ / 35

**Section 3 – Overall rating of the publication**

| **#** | **Item** | **Guidance** | **Pts** |
| --- | --- | --- | --- |
| 16 | **Overall quality** | Based on all preceding questions, evaluate the overall quality of this website as a source of information about [SEARCH TERM]. Rate on a scale of 1 (very low quality) to 5 (very high quality). |  |

**Total DISCERN Plus score:** ______ / 80

**Exclusion criteria ticked on the chart at the time of rating**

| □ | Limited information on search topic (< one paragraph) |
| --- | --- |
| □ | Complete access restricted by password |
| □ | Repeated server unavailability |
| □ | Direct forwarding to other domains |

**Predefined inclusion and exclusion criteria (internal study protocol, specified before the search)**

|  | **Criterion** | **Where applied** |
| --- | --- | --- |
| I1 | Inclusion: substantive information (at least one paragraph of continuous text) on at least one of the target pediatric neurosurgical conditions | Screening of the result list; re-checked at rating (chart criterion 1) |
| I2 | Inclusion: freely accessible without registration or payment | Screening of the result list; re-checked at rating (chart criterion 2) |
| E1 | Duplicate or mirror pages (identical content under two URLs, or a page appearing in one language result list after having been captured in the other) | Screening of the result list |
| E2 | Purely technical content for professionals without disease information for laypersons | Screening of the result list |
| E3 | Non-text formats without explanatory text (video, image or download-only pages) | Screening of the result list |
| E4 | Pages behind paywalls or with repeated server unavailability | Screening of the result list; chart criterion 3 (server unavailability) |
| E5 | Forums or social media posts without a stable information page | Screening of the result list |
| E6 | Complete access restricted by password | Chart criterion 2 |
| E7 | Direct forwarding to another domain | Chart criterion 4 |

The complete list of criteria was fixed in the internal study protocol of the search series before the Google searches were run and was applied when the result lists were screened. The four criteria printed on the chart are the page-level criteria that were ticked on the chart at the time of rating. Pages that met both inclusion criteria and none of the exclusion criteria were retained; professional documents that an informed layperson can read (e.g. a clinical practice guideline) were not covered by criterion E2 and were kept. In the present study five of 280 records were excluded (three duplicates under E1, two paywalled pages under E4); no record met criterion E2, E3, E5, E6 or E7.

**Positional comparison of Section 2 of the DISCERN Plus chart (items 9-15) with Section 2 of the original DISCERN instrument**

Items are listed side by side by item number only. The two columns do not correspond conceptually; the conceptual relationship is given in the following table.

| **#** | **DISCERN Plus item (this study)** | **Original DISCERN item at the same position (Charnock et al. 1999)** |
| --- | --- | --- |
| 9 | **Consequences/risks of [SEARCH TERM]** | 9. Does it describe how each treatment works? |
| 10 | **When to contact a physician** | 10. Does it describe the benefits of each treatment? |
| 11 | **Treatment options** | 11. Does it describe the risks of each treatment? |
| 12 | **Risks of treatment** | 12. Does it describe what would happen if no treatment is used? |
| 13 | **Diagnostic procedure** | 13. Does it describe how the treatment choices affect overall quality of life? |
| 14 | **Quality of life / treatment setting** | 14. Is it clear that there may be more than one possible treatment choice? |
| 15 | **Pre- or post-treatment recommendations** | 15. Does it provide support for shared decision-making? |

Items 9-15 of the DISCERN Plus chart are content items on the condition searched for and are not direct rewordings of the original treatment-choice items; validation of the original DISCERN therefore does not extend to Section 2 of this chart. Original DISCERN items 10 (benefits of each treatment), 14 (more than one possible treatment choice) and 15 (support for shared decision-making) have no counterpart in the DISCERN Plus chart, so these aspects are not assessed by the DISCERN Plus total; conversely, DISCERN Plus items 10, 13 and 15 have no counterpart in the original instrument.

**Conceptual relationship between the original DISCERN Section 2 items and the DISCERN Plus Section 2 items**

| **Original DISCERN item (Charnock et al. 1999)** | **Closest DISCERN Plus item (this study)** | **Relationship** |
| --- | --- | --- |
| 9. Does it describe how each treatment works? | 11. Treatment options (and their mechanism of action) | Partial overlap: item 11 asks whether treatment options and their mechanism are described |
| 10. Does it describe the benefits of each treatment? | None | Not assessed by the DISCERN Plus chart |
| 11. Does it describe the risks of each treatment? | 12. Risks of treatment | Corresponds |
| 12. Does it describe what would happen if no treatment is used? | 9. Consequences/risks of the condition | Partial overlap: item 9 addresses the consequences of the condition, not explicitly those of withholding treatment |
| 13. Does it describe how the treatment choices affect overall quality of life? | 14. Quality of life / treatment setting | Partial overlap: item 14 also covers the treatment setting |
| 14. Is it clear that there may be more than one possible treatment choice? | None | Not assessed; item 11 scores the description of options, not whether the existence of alternatives is made explicit |
| 15. Does it provide support for shared decision-making? | None | Not assessed by the DISCERN Plus chart |
| No counterpart | 10. When to contact a physician | DISCERN Plus item without original counterpart |
| No counterpart | 13. Diagnostic procedure | DISCERN Plus item without original counterpart |
| No counterpart | 15. Pre- or post-treatment recommendations | DISCERN Plus item without original counterpart |
