# Supplementary material for: Assessing the quality of internet resources on pediatric neurosurgery for caregivers
Source: Childs Nerv Syst. 2026 Nov 5;42(1):412. doi: 10.1007/s00381-026-07510-7 (PMC13740078; doi:10.1007/s00381-026-07510-7)
Supplement: Supplementary file 2 [file 381_2026_7510_MOESM2_ESM.docx]

**Supplementary File S2**

**Supplementary Table S1. Sensitivity analyses addressing clustering of records within web domains and confounding of language by host type.**

| **Analysis** | **Unit of analysis** | **n** | **English vs German, DISCERN Plus** | **English vs German, JAMA** | **Host-type effect** |
| --- | --- | --- | --- | --- | --- |
| Primary analysis | Condition-website record | 275 | U = 11960.0, p < 0.001 | U = 11504.5, p = 0.002 | DISCERN Plus H = 14.22, p = 0.007; JAMA H = 63.35, p < 0.001 |
| One record per domain (first occurrence) | Web domain | 148 | U = 3412.5, p = 0.009 | U = 3465.0, p = 0.004 | DISCERN Plus H = 12.44, p = 0.014; JAMA H = 42.76, p < 0.001 |
| Domain-level mean scores | Web domain | 148 | U = 3495.0, p = 0.003 | U = 3588.5, p < 0.001 | Not applicable |
| Mixed model, language only (random intercept for domain) | Record within domain | 275 | +5.60 points (95% CI 2.15 - 9.04), p = 0.001 | +0.47 benchmarks (95% CI 0.18 - 0.77), p = 0.001 | Not included |
| Mixed model, language and host type | Record within domain | 275 | +4.62 points (95% CI 1.12 - 8.12), p = 0.010 | +0.50 benchmarks (95% CI 0.24 - 0.75), p < 0.001 | DISCERN Plus: governmental +10.58 (3.81 - 17.35), p = 0.002; JAMA: educational +1.05, governmental +1.17, medical news +0.98 (all p < 0.001) vs university/medical center |
| Excluding governmental sites | Condition-website record | 256 | U = 10092.0, p = 0.001 | U = 9607.0, p = 0.014 | Not applicable |
| Within host type (educational / university or medical center / interest group-NGO / medical news) | Condition-website record | 59 / 137 / 38 / 22 | p = 0.001 / 0.073 / 0.78 / 0.121 | p = 0.125 / <0.001 / 0.299 / 0.041 | Not applicable |

U, Mann-Whitney U statistic; CI, confidence interval. Mixed models were fitted with a random intercept for web domain (148 domains); coefficients denote the difference for English-language compared with German-language pages and for the respective host type compared with university/medical-center pages. Within-host comparisons are reported in the order educational / university or medical center / interest group-NGO / medical news. No web domain contributed records in both languages, so each domain carries a single language in the domain-level analyses. The linear mixed model for the JAMA score (a bounded count of 0-4) is an approximation and served as a sensitivity analysis alongside the non-parametric tests. Within-host comparisons are exploratory; at a Bonferroni-corrected threshold for the eight tests (p < 0.006), only the educational-portal DISCERN Plus difference and the university/medical-center JAMA difference remain significant.

**Supplementary Table S2. Proposed pre-publication checklist for institutional patient information pages (preliminary proposal requiring prospective evaluation).**

| **No.** | **Checklist item (answer yes or no)** | **Derived from** |
| --- | --- | --- |
| 1 | Named author or authors with professional qualification and institutional affiliation | JAMA: authorship |
| 2 | Sources cited for factual and numerical statements, with links or full references | JAMA: attribution |
| 3 | Site ownership, funding and any commercial interest or sponsorship declared | JAMA: disclosure |
| 4 | Date of creation and date of last review displayed, with a planned review interval of no more than 24 months (a) | JAMA: currency |
| 5 | Aim of the page stated and the intended audience (caregivers of affected children) named | DISCERN Plus items 1-3 |
| 6 | All relevant management options described, including conservative management or watchful waiting where applicable (b) | DISCERN Plus item 11 (description of treatment options); original DISCERN item 14 (b) |
| 7 | Benefits, risks and expected outcomes described, including the consequences of the untreated condition (b) | DISCERN Plus items 9, 12 and 14; original DISCERN item 10 for benefits (b) |
| 8 | Areas of uncertainty or limited evidence explicitly acknowledged | DISCERN Plus item 8 |
| 9 | Plain language used (short sentences, technical terms explained, target reading level of grade 8 or lower), supported by a glossary or illustrations where helpful | EQIP |
| 10 | Support and contact options offered (department contact, patient organization, guidance on when to seek medical attention) | EQIP; DISCERN Plus items 7 and 10 |

Derived from the JAMA benchmarks, the reliability items (Section 1) and condition-specific content items (Section 2) of the DISCERN Plus chart (Supplementary File S1), the treatment-choice items of the original DISCERN and the EQIP instrument for written patient material. Proposed preliminary threshold before publication: all four transparency items (1-4) and at least four of the six content items (5-10) answered with yes; re-application at every review cycle. Neither the threshold nor the 24-month review interval was validated in the present study; both are intended as a starting point for prospective evaluation. (a) The 24-month interval is a pragmatic choice and not an evidence-based threshold. It follows institutional patient-information policies of health-care providers in the United Kingdom, which require clinical patient information to be reviewed every two years (e.g. Sherwood Forest Hospitals NHS Foundation Trust, patient information development and distribution policy GV-011) or at least every three years (NHS website content policy); the shorter interval was chosen because web pages can be updated more easily than printed leaflets. (b) The explicit presentation of alternatives including watchful waiting (item 6) and of treatment benefits (item 7) was not assessed in the present study: the DISCERN Plus chart scores whether treatment options, risks of treatment, consequences of the condition and effects on quality of life are described (items 9, 11, 12 and 14), but it does not contain the original DISCERN items on benefits of treatment (item 10), on the existence of more than one treatment choice (item 14) or on support for shared decision-making (item 15). These elements of checklist items 6 and 7 are therefore taken from the original DISCERN and from EQIP and are recommendations not supported by our own data.

**Supplementary Figure S1. DISCERN Plus scores by host type and language.**

Box plots show mean DISCERN Plus scores (16-80) for each host type separated by language; the number of records per group is given below each box. The language difference persisted in a linear mixed model with a random intercept for web domain and host type as a fixed effect (English vs German +4.62 points, 95% CI 1.12-8.12, p = 0.010). Governmental sites were almost exclusively English-language and are therefore shown for completeness only.
